# Draft Genome Sequences of *Agrobacterium nepotum* Strain 39/7^T^ and *Agrobacterium* sp. Strain KFB 330 

**DOI:** 10.1128/genomeA.00331-15

**Published:** 2015-04-23

**Authors:** Nemanja Kuzmanović, Joanna Puławska, Anđelka Prokić, Milan Ivanović, Nevena Zlatković, Katarina Gašić, Aleksa Obradović

**Affiliations:** a University of Belgrade–Faculty of Agriculture, Belgrade, Serbia; b Research Institute of Horticulture, Pomology Division, Skierniewice, Poland; c Institute for Plant Protection and Environment, Belgrade, Serbia

## Abstract

Tumorigenic strains of *Agrobacterium* spp. are responsible for crown gall disease of numerous plant species. We present here draft genome sequences of nonpathogenic *Agrobacterium nepotum* strain 39/7^T^ (CFBP 7436^T^, LMG 26435^T^), isolated from crown gall tumor on *Prunus cerasifera*, and tumorigenic *Agrobacterium* sp. strain KFB 330 (CFBP 8308, LMG 28674), isolated from galls on raspberry.

## GENOME ANNOUNCEMENT

The genus *Agrobacterium* comprises Gram-negative, predominantly soil-inhabiting bacteria. Tumorigenic strains contain conjugative tumor-inducing (Ti) plasmid in their genome and may cause crown gall disease of numerous plant species. The taxonomy of *Agrobacterium tumefaciens* (biovar 1) is still not fully resolved, since it is not a homogenous species but one composed of at least 11 genomic species (G1 to G9, G13, and G14). Therefore, it was proposed that they should be collectively called the *A. tumefaciens* species complex until all of them are formally named (1, 2). Although genomic species G2 and G14 were originally described as species *Rhizobium pusense* (3) and *Rhizobium nepotum* (4), respectively, they were recently renamed *Agrobacterium pusense* and *Agrobacterium nepotum* by Mousavi et al. (5). Here, we report draft genome sequences of *A. nepotum* strain 39/7^T^ (CFBP 7436^T^, LMG 26435^T^) and *Agrobacterium* sp. strain KFB 330 (CFBP 8308, LMG 28674).

Nonpathogenic strain 39/7^T^ was isolated from a crown gall tumor on *Prunus cerasifera* in Hungary in 1989 (4), while tumorigenic strain KFB 330 was isolated from a raspberry tumor in Serbia in 2012. Total genomic DNA of bacterial strains was extracted according to the protocol described by Aljanabi and Martinez (6). The genome sequencing was performed using 125-bp paired-end reads by an Illumina HiSeq2500 platform, and a total of 3,418,084 (39/7^T^) and 3,822,853 (KFB 330) paired-end reads were generated (BaseClear, Netherlands). After quality-control filtering and trimming, a *de novo* assembly was performed using CLC Genomics Workbench version 7.0.4, resulting in 79 (39/7^T^) and 74 (KFB 330) contigs. The genome coverage was 88.7× (39/7^T^) and 76× (KFB 330). The draft genome sequence of strain 39/7^T^ consisted of 5,328,872 bp, with an average GC content of 59.13% and an *N*_50_ lenth of 219,533 bp, while that of strain KFB 330 consisted of 6,298,483 bp, with an average GC content of 58.8% and an *N*_50_ length of 264,849 bp. The genome sequences were annotated by the NCBI Prokaryotic Genomes Automatic Annotation Pipeline (PGAAP). A total of 4,811 coding DNA sequences, 43 tRNAs, and 3 rRNAs were predicted for strain 39/7^T^, while 5,594 coding DNA sequences, 45 tRNAs, and 3 rRNAs were predicted for strain KFB 330.

The *telA* gene for protelomerase was detected in both strains sequenced, suggesting the presence of linear chromosome (chromid) in their genomes, which is the characteristic of the genus *Agrobacterium* (7). Multilocus sequence analysis (MLSA) based on *atpD*, *glnA*, *gyrB*, *recA*, and *rpoB* housekeeping loci revealed that strain KFB 330 represents a separate phylogenetic lineage within the genus *Agrobacterium*. This strain clustered with members of the *A. tumefaciens* complex but was clearly different from all known genomic species. A BLAST search (8) indicated the presence of Ti plasmid sequences in the genome of strain KFB 330, similar to those of nopaline-type pTiC58 (NC_003065) and pTi-SAKURA (NC_002147). The genome sequences reported here will serve as valuable references for studying taxonomic relationships and genetic characteristics of the genus *Agrobacterium*.

### Nucleotide sequence accession numbers. 

These whole-genome shotgun projects have been deposited at DDBJ/EMBL/GenBank under the accession numbers JWJH00000000 and JWIT00000000 for *A. nepotum* strain 39/7^T^ and *Agrobacterium* sp. strain KFB 330, respectively. The versions described in this paper are the first versions.
